# Helicopter parenting during emerging adulthood: Consequences for career identity and adaptability

**DOI:** 10.3389/fpsyg.2022.886979

**Published:** 2022-09-21

**Authors:** Joshua E. LeBlanc, Sean T. Lyons

**Affiliations:** ^1^Department of Human Resource Management and Labour Relations, Dhillon School of Business, University of Lethbridge, Lethbridge, AB, Canada; ^2^Gordon S. Lang School of Business and Economics, University of Guelph, Guelph, ON, Canada

**Keywords:** emerging adulthood, career adaptability, career identity, helicopter parenting, parenting profiles

## Abstract

This study explores the relationship between parental over involvement and the career development of emerging adults. Specifically, it investigates how emerging adults’ career meta competencies of vocational identity formation and career adaptability relate to perceived helicopter parenting. Participants included 491 emerging adults studying in a Canadian University (74.1% female, average age = 20.4 years old). We begin by reviewing the commonalities between helicopter parenting and other parenting constructs and styles. Next, using structural equation modeling, we explore the relationships between perceived helicopter parenting and the components of vocational identity (exploration: in depth exploration, in breadth; commitment: career commitment, identification with commitment; and reconsideration: career self doubt, career flexibility) and career adaptability, as well as the relationships between identity components and career adaptability. Third, we explore the association between perceived helicopter parenting and identity status progress (i.e., achievement, foreclosure, moratorium, undifferentiated, and searching moratorium). Results indicate that individuals reporting higher levels of perceived helicopter parenting experience significantly lower levels of career adaptability and in-depth exploration. Furthermore, these individuals report higher levels of career self doubt and are more likely to be in the vocational identity status of searching moratorium. Limitations and future research directions are discussed.

## Introduction

Parental involvement is essential to children’s social, cognitive, and emotional growth. Nearly all major career choice and development theories, both contemporary and traditional, recognize the contribution of parents on the career development of their children (Fouad et al., 2010; Peluchette et al., 2013; Patton et al., 2014). However, in recent years the involvement of parents has continued to rise, giving way to concerns that some parents are applying overly involved and developmentally inappropriate tactics to their children. As a result, we have seen reports that many of today’s emerging adults (18–25 year olds) are highly attached to and dependent on their parents (Segrin et al., 2012; Bradley-Geist and Olson-Buchanan, 2014; van Ingen et al., 2015). This phenomenon has raised concerns about parental coddling and the lack of independence and resilience it engenders in emerging adults (Bradley-Geist and Olson-Buchanan, 2014).

The role of parents in emerging adulthood differs from that of adolescence. Arnett (2014) argues that the feeling of not associating directly with either adolescence or adulthood is firmly rooted in one’s changing relationship with parents. For emerging adults, the once tentative vocational exploration of part-time employment is replaced by more serious exploration with greater implications toward one’s future career (Arnett, 2000, 2014). Furthermore, the communications that parents once had with their children regarding the importance of work are now reinforced or challenged by personal work experiences, observations or communication with other working adults (Bryant et al., 2006; Porfeli et al., 2008). Even though emerging adults may receive career guidance from a variety of sources, they continue to report that parents are a major source of both career and educational guidance (Mortimer et al., 2002; Fouad et al., 2010) and a primary outlet for discussing career issues (Otto, 2000). In light of this responsibility, parents may serve as a resource or hindrance to the career decision making of their offspring, particularly as they navigate the impending school-to-work transition in a challenging labor market (Parola and Marcionetti, 2021). While parents are closer to the labor market and thus may be perceived as a resource, emerging adults’ perceptions of parental interference are problematic when it comes to making career-related decisions (Parola et al., 2022).

### Over-parenting and the “helicopter parent”

A primary responsibility of parenting is to protect one’s children from harm, particularly from birth to early adolescence (Power and Hill, 2008). As children develop and levels of helplessness and immaturity dissipate, parental protectiveness and control should decrease accordingly. Previous protective behaviors once deemed adaptive, such as protecting children from dangerous objects or activities (Mayes et al., 2006), harmful media coverage (Hughes et al., 2006) or potentially upsetting family information (Lehman and Koerner, 2002) can pose immediate or eventual harm to children, if behaviors persist through development, and particularly into adulthood (Nelson, 2010). Context and the child’s stage of development ultimately dictate the appropriateness of parental behaviors. In other words, parental involvement *via* direction and affection should coincide with actual environmental dangers (Thomasgard and Metz, 1993; Wolf et al., 2009; Locke et al., 2012). When parental involvement exceeds the environmental risks and/or individual vulnerabilities of the child (e.g., physical or cognitive disability), over-parenting (OP) takes place (Ungar, 2009).

While there is no well-researched or agreed upon definition of OP (Locke et al., 2012), there are several distinguishable features. First, OP incorporates high levels of parental intrusion, removal of obstacles and encouragement of age-inappropriate dependence on parents (Segrin et al., 2012). Second, as Ungar (2009) describes, OP consists of excessive parental concern coupled with reduced flexibility at levels inconsistent with the safety of the environment or maturity of the child. Third, conceptualizations of OP tend to include high demand for child success (Locke et al., 2012), which can include instructions for the child on how to think, feel (Cooper-Vince et al., 2014) and act, with monitoring occurring both inside and outside the home (Morrongiello, 2005). Such monitoring includes variation in autonomy-limiting behavioral, and/or psychological control. Behavioral control refers to control over everyday activities (e.g., homework, chores), whereas psychological control involves manipulation of thoughts, feelings and emotions held toward parents (e.g., inducement of guilt, display of shame, possessiveness, withdrawal of affection; Barber, 1996).

In recent years, a distinct form of OP has received substantial attention in popular press and mass media under the colloquial label of helicopter parenting (Bradley-Geist and Olson-Buchanan, 2014). Coined by Charles Fay and Foster Cline, the term helicopter parent is said to represent the parenting style of some Baby Boomers (born 1946–1964) on their millennial children (Cline and Fay, 2006). Extending beyond the millennial generation, scholars describe this parenting style as unique to contemporary emerging adulthood in general (Padilla-Walker and Nelson, 2012; Luebbe et al., 2018). Segrin et al. (2012) define helicopter parenting as a version of over parenting in which “parents demonstrate excessive involvement in their children’s lives and apply developmentally inappropriate parenting tactics by failing to allow for levels of autonomy suitable to their child’s age” (p. 238). Luebbe et al. (2018) add that helicopter parenting may be a concise way to conceptualize multiple parenting behaviors particularly germane to the experiences of emerging adults as they transition to independence (p. 13). Thus, helicopter parenting is a distinct form of over parenting that takes place primarily during the life stage of emerging adulthood.

While the behaviors of helicopter parents reflect aspects of other forms of parenting described in the extant literature, this form of parenting is unique in several ways (LeMoyne and Buchanan, 2011; Schiffrin et al., 2014; Luebbe et al., 2018). In relation to other parenting constructs, initial studies consider helicopter parenting to be conceptually similar to an authoritarian parenting style, possessing a conformity orientation, and over-solicitous parenting found in infancy and early childhood (Segrin et al., 2012; Odenweller et al., 2014), and only moderately related to OP (Luebbe et al., 2018). However, despite possessing many of the same general dimensions (i.e., high warmth, high control, low autonomy granting, behavioral control), there is no indication that helicopter parents engage in psychological control; a defining tenet of OP, authoritarian parenting and conformity orientation (Odenweller et al., 2014; Luebbe et al., 2018). Furthermore, while helicopter parenting may be most conceptually similar to over solicitous parenting (Padilla-Walker and Nelson, 2012), it differs in two distinct ways: parenting context and parental responsiveness. In regard to context, over-solicitous parenting predominantly coincides with early childhood, from several months of age to 5 years in age (e.g., Rubin et al., 1999; Mark et al., 2002), often within clinical populations (e.g., social withdrawal/shyness: Rubin et al., 1997; anxiety disorders: Degnan and Fox, 2007). Meanwhile, helicopter parenting is distinct to contemporary emerging adulthood (Padilla-Walker and Nelson, 2012; Luebbe et al., 2018) and has been reported in non-clinical university samples as well (e.g., LeMoyne and Buchanan, 2011; Fingerman et al., 2012; Reed et al., 2016). Regarding responsiveness, research indicates over-solicitous mothers are highly unresponsive to bids for attention from their children (Rubin et al., 1997) while helicopter parents are highly responsive to the needs and demands of their adult children (Fingerman et al., 2012). Therefore, although both over-solicitous and helicopter parents directly intervene in their child’s behaviors, helicopter parents do so in a warm and affectionate manner that adheres directly to the child’s needs (whether real or perceived).

High levels of warmth/support are prioritized with helicopter parents (Padilla-Walker and Nelson, 2012). Coupling the presence of behavioral control with high levels of warmth and support represents a unique pattern of parenting dimensions. Additionally, helicopter parenting does not correlate significantly with other forms of parenting, such as permissive parenting, authoritative parenting or possessing a conversation orientation (Segrin et al., 2012; Odenweller et al., 2014). Further aspects of helicopter parenting that are not well captured in other parenting constructs include: a strong preoccupation with the child’s happiness; an inherent desire to solve the child’s problems; a perceived duty to protect the child from risk; and difficulty in trying to parse out parental goals and feelings from those of the child (Segrin et al., 2012).

### Career meta-competencies: Adaptability and identity

One of the issues of concern for researchers and practitioners alike is how contemporary emerging adults approach their careers. In a labor market that has become increasingly uncertain, competitive and fragmented (Baruch and Bozionelos, 2011), these changes have resulted in the use of new and contemporary career concepts (Gubler et al., 2014), requiring individuals to take on a self-directed role in career development. Amid this career context, a new set of career meta-competencies is required that focus on adaptability and a strong sense of identity (Gubler et al., 2014). According to Porfeli and Savickas (2012), the construct of career adaptability is composed of four components: concern, control, curiosity and confidence. Concern (planning) is the extent to which an individual is oriented toward and focused on preparing themselves for the future. Control (self-directedness) addresses the degree of self-discipline and drive toward making responsible decisions. Curiosity (exploration) refers to an open exploration of personal circumstances and seeking out of both career related information and opportunities. Confidence (self-efficacy) refers to one’s degree of belief that they possess the skills to overcome obstacles when they arise. Individuals may draw upon the four components of career adaptability when unfamiliar, complex or vague situations arise during career development, work transitions or traumatic work events (Porfeli and Savickas, 2012). As such, the four components of career adaptability may be considered essential competencies for emerging adults to develop as they proceed with their career development.

The other major contemporary career competency, vocational identity, involves career exploration behaviors, commitment to a vocational path, and the capacity for reconsideration of one’s choices in light of new circumstances and information (Porfeli et al., 2011). High levels of career exploration means that an emerging adult broadly learns about themselves and the work roles that may be suitable to pursue, develops work skills, and experiments with various jobs (i.e., in-breadth exploration). It also means that they engage in self and environmental exploration directed at a particular career and its requirements (i.e., in-depth exploration) (Savickas, 2002). Career commitment means having arrived at a career decision (i.e., commitment making) and having a sense of security and/or satisfaction with that choice (i.e., identification with commitment) (Luyckx et al., 2010). Finally, reconsideration comprises one’s ability to adjust their career path as a consequence of new work experiences, or shifts in interests, goals and values (i.e., flexibility), as well as the extent to which one experiences uneasiness, doubt or worry about their current career choice (i.e., career self-doubt) (Porfeli et al., 2011).

Building on Marcia’s (1966) foundational work on identity status, research on vocational identity typically scores individuals on each of its three components (Career Exploration, Career Commitment, and Career Reconsideration) and uses those scores to place individuals in one of six identity statuses (shown in Table 1). In addition to the four statuses proposed by Marcia (i.e., diffusion, moratorium, foreclosure and achievement), new statuses have been introduced to incorporate the dimension of reconsideration. More specifically, the *undifferentiated* status is incorporated from the work of Luyckx and colleagues (Luyckx et al., 2006) and a *searching moratorium* status from the work of Meeus and Crocetti (Crocetti et al., 2008a,b; Crocetti et al., 2009). The additional dimensions are particularly salient when talking about emerging adulthood, because they allow researchers to better explore the dynamic nature of identity development for individuals who cycle between statuses (Porfeli et al., 2011) and the identity crisis inherent to early adolescence and early adulthood (Crocetti et al., 2008b; Crocetti et al., 2009).

**TABLE 1:** Summary of identity status composition by dimension scores.

	Exploration		Commitment		Reconsideration	
	In-depth	In-breadth	Commitment making	Identification w/commitment	Self-doubt	Flexibility
Identity achieved	High	High	High	High	Low	Low
Foreclosure	Low	Low	High	High	Low	Low
Moratorium	Low	High	Low	Low	Avg	High
Identity diffusion	Low	Low	Low	Low	High	High
Searching Moratorium	High	High	High	High	High	High
Undifferentiated	Avg	Avg	Avg	Avg	Avg	Avg

### The consequences of helicopter parenting for career adaptability and identity

**FIGURE 1:**
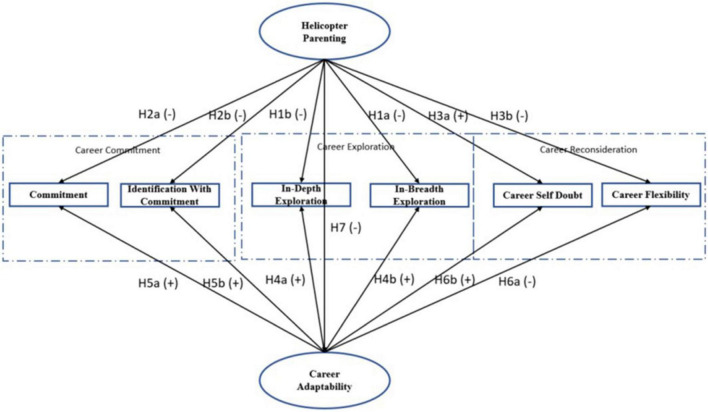
Conceptual model of hypothesized relationships between study variables.

We hypothesize that emerging adults’ perception of helicopter parenting relationships with their parents will be associated with their vocational identity status, both directly and *via* its impact on career adaptability. Our conceptual model is presented in Figure 1. In the following sections we discuss the hypothesized relationships within this model.

### Helicopter parenting and vocational identity status

Despite being paid substantial attention by the popular press, the helicopter parenting phenomenon is not well understood, particularly in regard to adult child preparation for the workplace (Bradley-Geist and Olson-Buchanan, 2014). What we do know, however, is that the presence of a helicopter parent can be deleterious toward identity development. For example Hong et al. (2015) found that higher levels of helicopter parenting are associated with greater amounts of procrastination and that procrastination negatively predicts performance monitoring, goal setting and pursuing goals. Bradley-Geist and Olson-Buchanan (2014) found that emerging adults with helicopter parents possess a reliance/dependence on others to find solutions for them when faced with workplace scenarios, as well as possess maladaptive work behaviors (inability to meet deadlines or engage in job seeking). van Ingen et al. (2015) found that children of helicopter parents report low levels of general self efficacy and high levels of peer mistrust, alienation, and poor peer communication. In a study of parent-child dyads, Segrin et al. (2012) found children reporting high levels of helicopter parenting showed elevated levels of entitlement. Konrath et al. (2014) argue that recent changes in family structure and parenting styles have led emerging adults to focus more on self-image and individuality than intimate or caring relationships with others.

An extended or disjointed period of exploration represents developmental delay, given that one of the fundamental developmental tasks of emerging adulthood is identifying and refining career choices (Buhl, 2007; Arnett, 2014). Dependence on one’s family may lead to not only inadequate exploration prior to finding a career but inadequate seeking and acquisition of career knowledge as well (Hardin et al., 2006). Such effects can lead to a form of career unreadiness referred to as career myth, wherein an individual exaggerates not only conceptual understanding of what a career means but the obligations inherent in a career as well (Cheung et al., 2014). Helicopter parenting may also limit the frequency and duration of time that emerging adults spend forming and refining network connections (social and professional) because they are under the impression that one or both parents will help them to secure an occupation. Helicopter parents may also disrupt the leisure activities of their adult children. Leisure provides a context for 18- to 25-year olds to freely explore the work context without being constrained by parents or formal work roles (Arnett, 2004). The greatest well being is experienced when an individual self-initiates and endorses an activity, not when it is imposed by others (Ryan and Deci, 2000). Seeking activities because they are perceived to be valued by someone else (i.e., parents) leads to increased stress, which is enhanced in complex and unpredictable environments (Shulman et al., 2014). With these factors considered, we make the following hypotheses:

**H1a: Perceived helicopter parenting will be negatively associated with in-breadth career exploration.**

**H1b: Perceived helicopter parenting will be negatively associated with in-depth career exploration.**

In addition to career exploration, high levels of career commitment are deemed essential in making progress toward identity achievement status (Marcia, 1966, 1993; Crocetti et al., 2008a,b; Luyckx et al., 2010; Porfeli et al., 2011). To possess high levels of career commitment one needs to have arrived at a decision (commitment making) and feel a sense of security and/or satisfaction with that choice (identification with commitment) (Luyckx et al., 2010). According to structural family theory (Minuchin, 1974), families that are excessively close or extremely disengaged provide little to no feelings of belongingness and/or personal autonomy. The hindrance of autonomy development by over-parenting has been proposed to lead emerging adults down a path of dependence on parents or other adult authority figures (Bradley-Geist and Olson-Buchanan, 2014) and reduced ability to accept responsibility (Segrin et al., 2012).

If dependence on parents and projection of responsibilities on others becomes pervasive, an emerging adult may develop a decision-making strategy coined *dependent* by Harren (1979). Unlike independent decision making skills (Johnson, 1978; Harren, 1979), which are beneficial in myriad situations, dependent decision making is maladaptive in both school and work conditions. Commitment to a career choice occurs most readily for those emerging adults who are both independent from and securely attached to their parents (Blustein et al., 1991) and because the children of helicopter parents do not embody such characteristics, there is heightened risk for career indecision, career unreadiness, uninformed or misinformed expectations, possessing an external locus of control, and dependent style thinking. Furthermore, emerging adults who perceive their parents to be helicopter parents will likely follow the suggestions of their parents and thus are perhaps less likely to assume ownership for decisions or attribute performance accomplishments to their own skills and abilities. With these factors considered, we make the following hypotheses:

**H2a. Perceived helicopter parenting will be negatively associated with career commitment making.**

**H2b. Perceived helicopter parenting will be negatively associated with identification with career commitment.**

As a consequence of learning and new work experiences, there will be inevitable shifts in one’s interests, goals and values (Porfeli et al., 2011). An ability to adjust a career path to fit these changes or vice versa, is central to the notion of career commitment flexibility. Individuals high in career commitment flexibility tend to engage in more in-breadth exploration, have high levels of career doubt and low levels of both career commitment and identification with career commitment (Porfeli et al., 2011). High levels of career doubt have subsequently been tied to increased likelihood of one experiencing an identity crisis (Kidwell et al., 1995; Crocetti et al., 2008b) and/or being in moratorium or diffused identity states (Porfeli, 2008). Furthermore, those in moratorium and diffusion experience greater career doubt than those in foreclosure or identity achievement (Porfeli et al., 2011).

The degree of structure instilled by parents can be detrimental to emerging adult career development when the environment is either over structured or under structured (Flum and Blustein, 2000). In the case of helicopter parenting, the social context is highly over structured and thus detrimental to development in several ways. A sub-optimal degree of structure undermines personal agency, inhibits exploration and does not provide clear expectations or sufficiently clear boundaries of appropriate behavior (Ryan and Deci, 2000). It is proposed here that perceived helicopter parenting and the over-structured environment it encompasses, instills high levels of career self doubt and lower levels of career commitment flexibility. In support of this proposition, Crocetti et al. (2008b) found that early (10–13 years old) and middle (14–19 years old) adolescents who perceive their parents as exerting high levels of psychological control and/or low levels of trust, engage in higher levels of reconsideration. It is unclear, however, if this relationship holds for perceived helicopter parenting, given its independence from psychological control (Padilla-Walker and Nelson, 2012). With these factors considered, we make the following hypotheses:

**H3a. Perceived helicopter parenting will be positively associated with career self-doubt.**

**H3b. Perceived helicopter parenting will be negatively associated with career flexibility.**

### Vocational identity status and career adaptability

The relationships between the career meta competencies of vocational identity status and career adaptability have been explored in extant literature. Therefore, it is our intention in this paper to be able to replicate those findings. For example, Porfeli and Savickas (2012) found evidence of concurrent validity between vocational identity and career adaptability. Research findings indicate that higher levels of adaptability correspond with greater amounts of exploration and commitment but lower levels of career reconsideration. Such characteristics are consistent with the profile of those in identity achievement. Porfeli and Savickas (2012) contend that low levels of adaptability may lead to feelings of anxiety or uncertainty when making career decisions, while high levels are indicative of more coherent/established identity. More explicitly, it is proposed that the relationship between adaptability resources and vocational identity is one where as the degree of commitment and certainty about career choices increases (diffused→ searching moratorium→ undifferentiated → moratorium → foreclosed → achieved), so do each of the four subscales of career adaptability.

This progression corresponds with the movement toward more advanced identity statuses. For example, previous studies have described foreclosed and diffused individuals (e.g., Skorikov and Vondracek, 1998; Kroger et al., 2010) as well as undifferentiated individuals (Porfeli et al., 2011), to be less advanced developmentally, compared to identity achieved individuals or those in searching moratorium. Porfeli and colleagues assert that such findings may be attributed to the dual effects of the dimensions of career reconsideration. More specifically, self-doubt tends to be predominantly negative in most contexts while flexibility tends to be predominately adaptive (Porfeli et al., 2011). In summary, increased adaptability, as a sign of vocational maturity, will be associated with the identity statuses attributed to maturity. Therefore, the following hypotheses are made:

**H4: Career adaptability will be positively associated with career exploration, both (a) in depth and (b) in-breadth career exploration.**

**H5: Career adaptability will be positively associated with career commitment, both (a) commitment making and (b) identification with career commitment.**

**H6a: Career adaptability will be negatively associated with career flexibility.**

**H6b: Career adaptability will be positively associated with career self-doubt.**

### Helicopter parenting and career adaptability

The capacity for an individual to make their own career choices has been and continues to be central to many theories and models of career decision-making (Inkson et al., 2015).

Numerous studies illustrate the deleterious effects OP and helicopter parenting can have on the personal agency of adult children (Blatt, 2004; Marano, 2004; Coburn, 2006; LeMoyne and Buchanan, 2011; Givertz and Segrin, 2012; Segrin et al., 2012). This presents a serious issue for the career actor as the exertion of personal agency is heralded as being more important than ever for career development (Hall, 2004; Briscoe et al., 2006). Major determinants of personal agency are the perspectives that an individual holds of him or herself in familial and social environments, termed competence or self-efficacy (Reed et al., 2016). Self-efficacy beliefs are formed and modified through one or more of the following: through performance accomplishments, *via* social persuasion, through vicarious learning or based on the physiological reactions one has to particular events (Lent and Brown, 1996). Because individuals launching a career for the first time may not have not had the ability to adequately develop career related self-efficacy beliefs, it is likely that emerging adults form and modify their self-efficacy beliefs primarily through social persuasion and vicarious learning from one or more parental figures.

In the context of family, self-efficacy is a culmination of the environment at home and the autonomy that it affords (Reed et al., 2016). The family context should provide emerging adults with what Kroger (2007) describes as an optimal level of accommodative challenge. What this means is that the environment should present the emerging adult with new life experiences/challenges that require a clear and sufficient use of autonomy in order to complete them successfully. Emerging adults that perceive their parents to be autonomy supportive are better able to adjust to the challenges of emerging adulthood (Chirkov and Ryan, 2001) due to the belief that their needs are being met. If the perception is that autonomy or other psychological needs (i.e., competence and relatedness) are not being met, the environment can be classified as a need frustrating environment (Reed et al., 2016). Helicopter parenting presents a need-frustrating environment as they have difficulty in *letting go* of their adult children (Coburn and Treeger, 2003). When parents “let go,” they allow their children to freely explore their new college or work environment, including making their own decisions and dealing with the consequences of their actions. By letting go, a parent helps to reduce the reliance an emerging adult may have on one or both parents when faced with challenging situations (Wolf et al., 2009). We hypothesize the following:

**H7. Perceived helicopter parenting will be negatively associated with career adaptability.**

## Materials and methods

### Participants and procedures

Participants included 491 emerging adults from a large university in Western Ontario, Canada. A majority of participants were not members of a visible minority group (75.2%), were female (74.1%), were single/never married (96.5%), lived in a rental apartment when enrolled in school (75.4%), resided with parents when not enrolled in school (79.2%), and had fewer than 2 siblings (91.5%). Participants were recruited from both the school of business (54%) and the college of biological sciences (46%). All years of registration were accounted for, from first year (0.6%) to second (27.5%), to third (29.9%), to fourth (28.7%), and even fifth year students (6.5%). The average age of participants was 20.4 years old. Students enrolled in graduate programs accounted for the remaining 6.7%. Regarding combined parental income, 5.9% reported under CAD $40K, 28.2% reported between $41,000 and $90,000, 38.5% reported between $91,000 and $150,000, and 27.5% reported over $150,000. Regarding parent education status, it was reported that mothers and fathers most commonly held Bachelor’s degrees (36.5 and 34%, respectively), while 14.3% of mothers and 21.2% of fathers held Master’s degrees or higher. Regarding emerging adult employment status we found the following: 173 unemployed (35.2%), 78 working fewer than 10 h a week (15.9%), 60 working 11–20 h per week (12.2%), 43 working 21 to 30 h per week (8.8%), and 137 working 31 or more hours per week (27.9%).

Participants completed a 29-question, 105-item survey, which took on average 13 min to complete. The survey was created, and data was collected, using an online survey platform (Qualtrics). Research ethics board approval was granted from the university. Participants were induced with a prize draw for one of 20, $10 university hospitality gift cards.

### Measures

#### Career adaptability

Career adapt-ability was measured using the 12 item *Career Adapt-Abilities Scale Short form* (CAAS-SF: Maggiori et al., 2017), which was adapted from the 24-item Career Adapt-Abilities Scale (CAAS: Savickas and Porfeli, 2012). The CAAS-SF has four, three-item scales measuring concern, control, curiosity and confidence. Items begin with the following statement: “Different people use different strengths to build their careers. No one is good at everything, each of us emphasizes some strengths more than others. Please rate how strongly you have developed each of the following abilities using the scale below” (Savickas and Porfeli, 2012). Sample items include: “thinking about what my future will be like (concern), “Making decisions about myself” (control), “Observing different ways of doing things” (Curiosity), and “Working up to my ability” (Confidence). Participants can respond from (1) not strong to (5) strongest. Measure reliability was found to be α = 0.79.

#### Vocational identity status

Vocational identity status was measured using the 30-item *Vocational Identity Status Assessment* (VISA: Porfeli et al., 2011). The VISA contains the three higher order dimensions (Career Exploration, Career Commitment, and Career Reconsideration) with two subscales per dimension. All VISA items were scored on a 5-point Likert scale of (1) strongly disagree to (5) strongly agree. Sample items include: “learning about various jobs that I might like” (In breadth exploration), “identifying my strongest talents as I think about careers” (In-depth exploration), “I know what kind of work is best for me” (career commitment making), “My career will help me satisfy deeply personal goals” (identification w/commitment), “I doubt I will be able to find a career that suits me” (career self-doubt), “My work interests are likely to change in the future” (career flexibility). Subscale reliability was found to be the following: Career Commitment α = 0.77; ID W/commitment α = 0.70; Career Self Doubt α = 0.80; Career Flexibility α = 0.85; Exploration in Depth α = 0.70; Exploration In-Breadth α = 0.83.

#### Perceived helicopter parenting

The degree of helicopter parenting participants perceived in their parental relationships was measured using the 15-item *Helicopter Parenting Instrument* (HPI; Odenweller et al., 2014). Items of the HPI are measured on a five-point Likert scale ranging from strongly disagree (1) to strongly agree (5) and higher average scale scores indicate greater prevalence of helicopter parenting. Items 5 and 14 are reversed scored. Sample items include: “My parent tries to make all of my major decisions,” and “My parent thinks it is his or her job to shield me from adversity.” Measure reliability was found to be α = 0.85.

### Analytic procedure

Analyses were conducted using version 26 of the Statistical Package for Social Sciences and the Analysis of Moment Structures (AMOS) statistical package. Data analysis consisted of a four-step process. First, data cleaning took place in order to meet the assumptions of the statistical procedures incorporating hypothesis testing [ANOVA, ANCOVA, covariance based Structural Equation Modeling (SEM)]. Tests were also conducted for common method bias (Harman, 1976), unengaged respondents and missing values.

Second, the measurement model of each latent construct (Career Adaptability, Vocational Identity, and Perceived Helicopter Parenting) was evaluated using confirmatory factor analysis. The testing of each measurement model is essential in understanding whether the observed/measured variables adequately represent the study constructs, and is thus foundational to assessing and interpreting the structural model (Jackson et al., 2009). To interpret the fit of the model, the following indices were used: the Chi Square value (CMIN); relative Chi-square statistic (CMIN/df); Root Mean Square Error of Approximation (RMSEA); Comparative Fit Index (CFI), Tucker-Lewis Index (TLI) and standardized root mean square residual (SRMR). Fit indices were tested against the suggested cut-offs by Hu and Bentler (1999) of 0.95 for CFI and TLI, less than 0.06 for RMSEA and less than 0.08 for SRMR.

Third, to test all hypotheses, a latent variable structural model measuring the relationship between perceived helicopter parenting, the 6 dimensions of vocational identity status and career adaptability was tested. Furthermore, the relationships between the dimensions of vocational identity status and career adaptability were also tested. Fit indices were once again tested against the suggested cut-offs by Hu and Bentler (1999). Sequential chi-square differences tests were used to evaluate competing nested models.

Fourth, to test for differences in perceived helicopter parenting by identity status progress, the statistical procedures of cluster analysis and analysis of covariance (ANCOVA) were used. A two-step cluster analysis procedure was used to determine group membership for the VISA statuses. This is a common procedure in research addressing vocational identity status, and extant studies continue to conclude that a six-cluster solution is most satisfactory (Crocetti et al., 2008a; Porfeli et al., 2011). In the first step, scores on each of the 6 identity sub-scales were standardized (*M* = 0, SD = 1) and the transformed scores of each subscale underwent K-means cluster analysis to define the number of clusters (i.e., levels of student response to each dimension). Given that research has consistently determined that six-clusters explains the greatest variance when clustering by all six dimensions of vocational identity, the number of clusters was specified to be six. Second, the k-means method determined a cluster number for each case/respondent according to distance from the cluster centers. Final output indicates the number of cases in each cluster as well as the similarities and differences among cluster profiles (e.g., final cluster centers and Euclidian distances between cluster centers). Analysis of covariance (ANCOVA) was then used to test for status differences in levels of perceived helicopter parenting, while controlling for the influence of covariates.

## Results

### Confirmatory factor analysis

The first series of analyses following data preparation included the testing of three measurement models using confirmatory factor analysis. For vocational identity, it was anticipated that the data would support a six-factor structure with all 30 indicators loading independently onto one of the six latent factors (i.e., exploration in-depth, exploration in breadth, career commitment, identification with commitment, career self doubt and career flexibility). Results support the conclusion that the six-factor model with covaried factor errors fit the data well (CMIN 890.73; CMIN/DF 2.31; RMSEA 0.051; CFI 0.91; TLI 0.904; SRMR 0.058; AIC 1048.73), adding further support to the work of Porfeli et al. (2011).

For career adaptability, it was anticipated that the data would best support a second-order model with all 12 indicators loading independently onto one of four first-order latent factors. Overall, CFA showed that the data fit the second order model well (CMIN of 228.29; CMIN/df of 4.66; RMSEA of 0.086; CFI of 0.86, TLI of 0.90, and SRMR of 0.067). Acceptable fit with the second order model is consistent with prior empirical studies (e.g., Savickas and Porfeli, 2012; van Vianen et al., 2012; Maggiori et al., 2017).

Thirdly, for perceived helicopter parenting, it was anticipated that data would support a single-factor structure with all 15 items loading independently onto the one latent factor. Results support the conclusion that the single-factor model with covaried factor errors fit the data well (CMIN of 200.684; CMIN/DF of 2.54; RMSEA of 0.056; CFI of 0.930; TLI of 0.906; SRMR of 0.049; and AIC of 282.68). Acceptable fit of the single factor structure is consistent with the work of Odenweller et al. (2014).

### Study hypotheses

**FIGURE 2:**
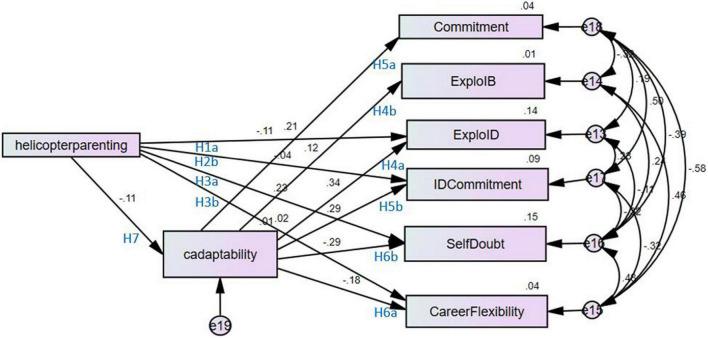
Final structural model.

Several structural models were tested, beginning with the theoretical model with all hypothesized paths estimated. Taking the suggestion of Byrne (2010), each subsequent model involved the addition or subtraction of a single parameter from the theoretical model. The final structural model is illustrated below (see Figure 2). The model fit the data well (CMIN 33.50; CMIN/DF 4.79; RMSEA 0.088; CFI 0.974; TLI 0.897; SRMR 0.052; AIC 91.504). Findings for the test of the hypothesized relationships between study variables are listed in Table 2. More specifically, support was found for: H1a, as perceived helicopter parenting was negatively associated with in-depth career exploration (β = −0.106, *p* < 0.01); H3a, as perceived helicopter parenting was positively associated with career self-doubt (β = 0.219, *p* < 0.001); H7, as perceived helicopter parenting was negatively associated with career adaptability (β = −0.110, *p* < 0.01); H4a, as career adaptability was positively associated with in-depth exploration (β = 0.343, *p* < 0.001); H4b, as career adaptability was positively associated with in-breadth exploration (β = 0.122, *p* < 0.01); H5a, as career adaptability was positively associated with career commitment (β = 0.207, *p* < 0.001, 7); H5b, as career adaptability was positively associated with identification with commitment (β = 0.298, *p* < 0.001); and H6b, as career adaptability was negatively associated with self-doubt (β = −0.292, *p* < 0.001). H6a was not supported, as career adaptability was negatively associated with career flexibility, not positively associated as hypothesized (β = −0.186, *p* < 0.001). Support was also not found for H1b (β = −0.027, *ns*), H2a (β = 0.041, *ns*), H2b (β = −0.011, *ns*) or H3b (β = −0.013, *ns*).

**TABLE 2:** Summary of standardized and unstandardized regression coefficients for significant results.

Path	Standardized estimate	Estimate	S.E.	C.R.	*P*
H1b. Helicopter Parenting –> Exploration ID	–0.106	–0.056	0.02	–2.618	0.01
H3a. Helicopter Parenting –> SelfDoubt	0.219	0.224	0.04	6.092	***
H4a. Adaptability –> Exploration ID	0.343	0.338	0.04	8.133	***
H4b. Adaptability –> Exploration IB	0.122	0.217	0.08	2.723	0.01
H5a. Adaptability –> CommitmentMaking	0.207	0.292	0.06	4.683	***
H5b. Adaptability –> Id w/Commitment	0.298	0.553	0.08	6.911	***
H6a. Adaptability –> Flexibility	–0.186	–0.355	0.09	–4.195	***
H6b. Adaptability –> SelfDoubt	–0.292	–0.56	0.08	–6.971	***
H7. Helicopter Parenting –> Adaptability	–0.11	–0.059	0.02	–2.459	0.01

***Refers to a significance value less than 0.001. S.E refers to standard error. C.R stands for critical ratio.

### K means cluster analysis

K means cluster analysis with Ward’s linkage and squared Euclidian distance was used to determine what homogenous clusters of emerging adults arose from the standardized scores on each of the dimensions of vocational identity status. The number of clusters was set to 6 to remain consistent with previous empirical findings and theoretical discussion regarding the number of potential identity statuses (Crocetti et al., 2008b; Porfeli et al., 2011). Results indicate that commitment *F*(5,485) = 147.155, *p* < 0.001, identification with commitment *F*(5,485) = 82.152, *p* < 0.001, self doubt *F*(5,485) = 139.120, *p* < 0.001, flexibility *F*(5,485) = 140.354, *p* < 0.001, exploration in breadth *F*(5,485) = 116.728, *p* < 0.001, and exploration in depth *F*(5,485) = 109.901, *p* < 0.001 are all important factors in determining cluster membership. Omnibus *F*-Tests indicate that clusters differ significantly by levels of commitment *F*(5,485) = 176.283, *p* < 0.00, identification with commitment *F*(5,485) = 104.660, *p* < 0.001, self-doubt *F*(5,485) = 137.393, *p* < 0.001, flexibility *F*(5,485) = 104.885, *p* < 0.001, exploration in-breadth *F*(5,485) = 38.060, *p* < 0.001, and exploration in-depth *F*(5,485) = 46.772, *p* < 0.001.

**FIGURE 3:**
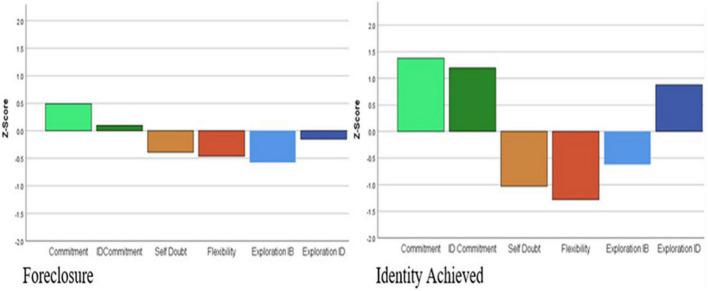
Profiles for advanced (foreclosure and identity achieved) identity statuses.

**FIGURE 4:**
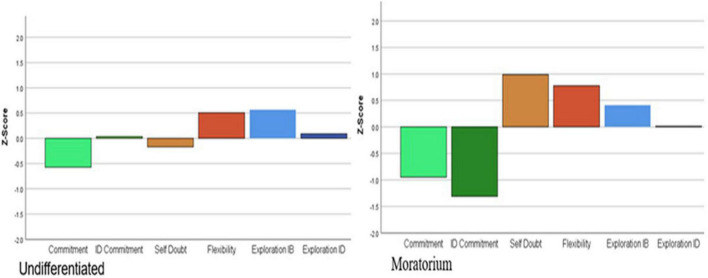
Profiles for moderate (undifferentiated and moratorium) identity statuses.

**FIGURE 5:**
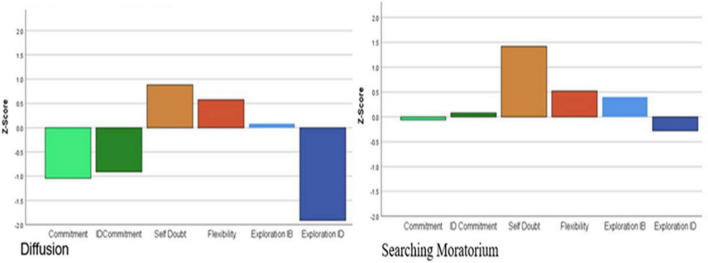
Profiles for delayed (diffusion) and mixed (searching moratorium) identity statuses.

Figures 3–5 illustrate the distribution of standardized factor scores by cluster membership. The determination of each cluster name was based on the summary of status dimensionality provided in Table 1. The percentages of emerging adults within each identity status were as follows: 15.9% were identity achieved, 9.4% in searching moratorium, 14.3% in moratorium, 27.5% in foreclosure, 5% were in diffusion, and 27.9% were undifferentiated.

Analysis of Variance (ANOVA) was run to test for differences in reported levels of helicopter parenting by cluster membership. There was a significant main effect of identity status, *F*(5,470) = 7.18, *p* < 0.001; partial η2 = 0.07. Bonferroni *post-hoc* comparisons were conducted to follow up on the omnibus findings. Results indicate that for identity status: (a) Undifferentiated individuals (*M* = 2.52, SD = 0.53) reported significantly lower levels of perceived helicopter parenting than individuals in searching moratorium (*M* = 3.13, SD = 0.51, *p* < 0.001), (b) Those in foreclosure (*M* = 2.69, SD = 0.54) reported significantly lower levels of perceived helicopter parenting than those in searching moratorium (*p* < 0.001), (c) Identity Achieved individuals (*M* = 2.57, SD = 0.58) reported significantly lower levels of perceived helicopter parenting than those in searching moratorium (*p* < 0.001), and (d) Those in searching moratorium reported significantly higher levels of helicopter parenting than individuals in moratorium (*M* = 2.71, SD = 0.58, *p* < 0.001).

### Identity status progress

Results from the cluster analysis and ANOVA were further distilled in order to explore the effects of perceived helicopter parenting on level of identity progress. Cluster categories were collapsed into delayed (diffusion), moderate (moratorium and undifferentiated), advanced (foreclosure and achievement) and mixed (searching moratorium) progress domains (see Porfeli et al., 2011). A series of one-way ANCOVAs were conducted to compare levels of perceived helicopter parenting by identity progress while controlling for SES, gender, year of registration and cohabitation statuses. Table 3 reports the estimated marginal means of perceived helicopter parenting for each of the identity status progress groups following the inclusion of covariates. The table also reports out the results of tests for between subject effects with each covariate considered.

**TABLE 3:** Summary of estimated marginal means and between subject effects with and without controlling for the effects of covariates.

	Identity status progress				Between subjects		
Covariates	Delayed	Moderate	Mixed	Advanced	*F*	partial η 2	Sig.
No Covariate	2.773	2.583	3.128	2.646	12.827	0.073	0.000
SES	2.765	2.586	3.118	2.646	12.222	0.070	0.000
Only Child	2.769	2.585	3.110	2.648	11.646	0.067	0.000
Gender	2.775	2.579	3.122	2.651	12.793	0.073	0.000
Year Reg	2.777	2.586	3.114	2.645	12.093	0.069	0.000
Res-Student	2.782	2.584	3.114	2.647	12.135	0.070	0.000
Res-Not Student	2.783	2.580	3.127	2.647	13.068	0.075	0.000

Outcome is helicopter parenting.

Results indicate levels of perceived helicopter parenting differed according to identity status progress even after controlling for the effects of covariates. More specifically, reported helicopter parenting differed by identity status progress groups after controlling for SES, *F*(3,486) = 12.22, *p* < 0.001, partial eta squared = 0.070, gender *F*(3,486) = 12.793, *p* < 0.001, partial eta squared = 0.073, year of registration *F*(3,486) = 12.093, *p* < 0.001, partial eta squared = 0.069, residence as a student *F*(3,486) = 12.135, *p* < 0.001, partial eta squared = 0.070, and residence when not a student *F*(3,486) = 13.068, *p* < 0.001, partial eta squared = 0.075. *Post-hoc* tests revealed that while each analysis yielded slight changes in mean differences between identity progress groups, significant differences in marginal means remained consistent. Namely, individuals with mixed identity progress reported significantly higher levels of perceived helicopter parenting compared to those with delayed, moderate or advanced progress.

## Discussion

This study investigated the association between perceived helicopter parenting and vocational identity status, both directly and *via* its relationship with career adaptability. Overall, findings indicate that perceived helicopter parenting may present barriers to the pursuit of a values-driven, self-directed protean career path. Participants reporting higher levels of helicopter parenting reported significantly lower levels of career adaptability and in-depth exploration. Furthermore, these individuals reported higher levels of career self doubt and were more likely to be in the vocational identity status of searching moratorium. Therefore, these individuals may be fluctuating between identity achievement and moratorium, perhaps due to high levels of flexibility and self-doubt. These findings challenge the notion that emerging adults are active agents in the self-construction of personal identity (Schwartz et al., 2005; Layland et al., 2018), by highlighting the relationship between parental interference (Dietrich and Kracke, 2009) and personal autonomy, motivation and career decision making self-efficacy (Guan et al., 2016). This may help to explain a lack of significance between perceived helicopter parenting and in-breadth exploration but a significant relationship with in-depth exploration. Extended exposure to non-self-led vocational activities may be associated with increased stress and strain, reducing self-regulatory capacities such as career adaptability (i.e., adaptability resources) (Johnston, 2018).

Career adaptability represents self regulatory resources that allow for more effective coping strategies to be enacted (Savickas, 1997; Guan et al., 2016). Because helicopter parenting is associated with low levels of career adaptability, it is likely that children will continue to exhibit maladaptive coping strategies. Repeated inability to meet educational or work-related challenges may lead to lowered willingness to explore career options, feelings of mastery (competence) and career related self-efficacy. In fact, this relationship is proposed by the career construction model of adaptation (Savickas and Porfeli, 2012; Savickas, 2013), which argues that adaptivity (e.g., personality, hope, optimism) positively affects career adaptability, which in turn affects adapting responses (e.g., career self-efficacy, career exploration) and eventually adaptation results (e.g., vocational identity, job satisfaction, engagement). Given that helicopter parenting is negatively associated with both in-depth exploration and career adaptability, it is not surprising that this study also found a significant relationship with vocational identity status.

The second aim of this study was to determine the relationship between perceived helicopter parenting and identity development. Identity achievement requires navigation of a period of crisis in which an individual explores and refines their values and interests (Marcia, 1966, 1980) into eventual commitments. It was hypothesized that increased levels of helicopter parenting would be positively associated with delayed (i.e., diffusion) and mixed (i.e., searching moratorium) vocational identity statuses, and negatively associated with moderate (i.e., moratorium, undifferentiated) and more advanced statuses (i.e., foreclosure and identity achievement). While bivariate correlations all matched the hypothesized direction, only two (HP and mixed, HP and moderate) were statistically significant. Furthermore, regression analysis indicated that only mixed identity progress was a significant predictor of reported helicopter parenting. ANCOVA revealed that those with mixed identity status progress continued to have the highest levels of helicopter parenting even after controlling for SES, gender, year of registration and cohabitation status (i.e., residence student, residence not student).

Of the six identity profiles found in this study, only searching moratorium had significant ties to perceived helicopter parenting. Searching moratorium represents a unique identity status. Individuals in searching moratorium experience both adaptive (e.g., high flexibility, exploration and commitment to a career path) and maladaptive (high self-doubt) elements (Porfeli et al., 2011). Particularly troublesome for this group is that high levels of career self-doubt have repeatedly been tied to increased likelihood of one experiencing an identity crisis (Kidwell et al., 1995; Crocetti et al., 2008b). This is likely due to the frequent fluctuation between achieved and moratorium statuses and the effects on well-being and full identity formation that such vacillation creates (Porfeli et al., 2011). Scholars have referred to this fluctuation between identity achievement and moratorium as the MAMA cycle (Moratorium-Achievement Moratorium- Achievement: Stephen et al., 1992).

MAMA cycles are more prevalent among adolescents or high school aged students (Crocetti et al., 2008b; Porfeli et al., 2011). At the end of adolescence (i.e., emerging adulthood), energy and attention are more strongly directed at fulfilling commitments and if MAMA cycles occur, they tend to be the result of major life events (e.g., loss of job or spouse) that trigger a loss of identity (Stephen et al., 1992). Therefore, the positive relationship between perceived helicopter parenting and searching moratorium suggests that this parenting style may promote identity disequilibria, or a continual charting of different vocational paths.

Helicopter parenting may lead to identity disequilibria by negatively affecting resilience during emerging adulthood. Studies of emerging adulthood highlight two factors that facilitate resilience. First are cognitive skills and abilities, such as general intelligence, the ability to plan ahead and moderation of personality traits along the dimensions of neuroticism and emotional stability (Burt and Paysnick, 2012; Arnett, 2014). Second, is the presence of a healthy relationship with at least one other person who expresses care for one’s well-being and who can offer support, guidance and resources if needed (Burt and Masten, 2010; Arnett, 2014). Results of this study indicate that perceived helicopter parenting is associated with lower coping resources of career adaptability, which are essential in navigating work transitions or traumas. In addition, studies have confirmed that helicopter parenting leads to reduced general coping efficacy, as well increased neuroticism (Odenweller et al., 2014). While helicopter parents are warm and responsive to their child’s needs, they act in developmentally inappropriate ways *via* protection from daily stressors and disappointments. Thus, helicopter parenting may detrimentally affect both factors that facilitate resilience in emerging adulthood. As a result, emerging adults may be emotionally unstable and highly susceptible to any negative career -related experiences, with even the smallest challenges being perceived as significant. When this occurs, individuals may be more likely to transition from achievement to moratorium.

Finally, this study tested the relationships between vocational identity and career adaptability. Significant positive relationships were found between adaptability and commitment (commitment and identification with commitment) as well as exploration (in-depth and in breadth exploration). Significant negative relationships were found between adaptability and the dimensions of career reconsideration (self-doubt and flexibility).

A strong body of literature helps to confirm the significant relationships found between identity and adaptability dimensions. For example, both longitudinal (e.g., Hirschi, 2009, 2012) and cross sectional (e.g., Creed and Patton, 2003; Savickas and Porfeli, 2012) studies have confirmed positive relationships between adaptability and both dimensions of exploration and commitment. Despite the reconsideration of commitment dimension being a relatively recent addition to the original exploration and commitment domains (see Porfeli et al., 2011), research supports the notion that higher levels of flexibility and lower levels of self-doubt contribute to greater career adaptability (Negru-Subtirica et al., 2015). Findings in the current study suggest a different pattern, with adaptability negatively predicting both reconsideration dimensions. This directionality also makes sense given that career adaptability is a measure of one’s ability to self regulate career related behaviors (Savickas, 1997). Thus, the readiness and capacity to master career related tasks should be antecedent to any career specific behavior. Upon reflection of vocational choices, it is then likely that career adaptability can be enhanced through flexibility and reduced through self-doubt.

The finding in this study that adaptability is associated with lower levels of flexibility should be explored. It may be that the commitment evaluation cycle for those high in adaptability is one of confidence. In other words, individuals scoring high on the global measure of career adaptability may have scored particularly high on the dimensions of control and confidence; resulting in the need for flexibility being perceived as less salient to them for the time being. As Porfeli and Savickas (2012) contend, low levels of adaptability may lead to feelings of anxiety or uncertainty when making career decisions, while high levels are indicative of more coherent/established identity. Therefore, higher levels of adaptability may reduce anxiety and uncertainty, leading to lower levels of self-doubt and the perceived need for flexibility. A university environment consisting of elevated levels of entitlement (Twenge, 2006) and postmodern thought (Smith et al., 2011), coupled with most students having little to no work experience, may mean that any vocational commitments are less likely to be reflected upon critically, if at all. The hypothesized relationship of higher levels of career adaptability being associated with higher levels of flexibility may only exist after an individual has significant first-hand work experience characterized by significant work transitions.

### Limitations and future research directions

The findings presented in this study need to be considered in light of several limitations. First, families are complex systems marked by high levels of variation and instability (Carlson and Meyer, 2014). The character, composition and resource sharing within families is affected by this level of complexity. Furthermore, the complexity of families and parent-child interactions is dynamic, and perhaps not fully captured using self-report measures. The findings of this study are based on self-reports, which may be subject to memory bias, response fatigue, social desirability responding, unengaged responses, and/or distortion based on symptomatology. Additionally, the self report items in this study considered only one perspective- that of the emerging adult. It would be more informative to assess both child and parent perceptions of parenting behavior. In other words the single study, single-perspective cross-sectional nature of this research is limited in its ability to understand complex family systems. Future research can build upon the findings reported here by using a longitudinal research design that tracks the perceptions of emerging adults, and their parents, over the school to work transition.

Second, helicopter parenting occurs when “parents demonstrate excessive involvement in their children’s lives and apply developmentally inappropriate parenting tactics by failing to allow for levels of autonomy suitable to their child’s age” (Segrin et al., 2012, p. 238). The current study relied on emerging adults’ perceptions of helicopter parenting behaviors on the part of their parents. We therefore could not make explicit the point at which parenting behaviors were perceived as developmentally detrimental. Future qualitative work may help to parse this out. Longitudinal quantitative work that observes actual parental behaviors over time and incorporates actual career outcomes (e.g., objective or subjective career success, securing full time employment) is also viable in addressing this research question. Research that explores how to best encourage emerging adults to assess levels of helicopter parenting in their own lives may help to address this as well. Tools such as My System of Career Influences-MSCI (McMahon et al., 2005) or the Family Influence Scale (FIS: Fouad et al., 2010), can help adult children to explore how parents have influenced their career and work choices, attachment bonds and ability to reflect upon past events. For non-clinical and clinical populations alike, merely reviewing the items in the helicopter parenting inventory might raise awareness. Perhaps classes in University about “adulting” or career readiness could encourage greater independence. Career seekers who are dependent on their helicopter parents may find it in their best interest to engage in a multiplicity of extracurricular and extra-occupational activities, many of which are offered in colleges and universities. Seeking membership in various groups or engaging in hobbies may help emerging adults to gain a greater sense of identity, experience shortcomings and subsequently learn coping mechanisms to help foster resilience later on. Research is needed that explores the efficacy of such programs and interventions on their ability to attenuate the effects of helicopter parenting on career adaptability and identity.

Similarly, research should explore the potential positive effects of healthy relationships with romantic partners, academic advisors, mentors, and other influential people. Resilience for emerging adults is in part shaped by the presence of a healthy relationship with at least one other person who expresses care and offers support (Burt and Masten, 2010). Such relationships were not explored in this research, despite the potential they have at moderating the effects of helicopter parenting on career adaptability. In regard to identity, Lambert et al. (2010) found that a “sense of meaning” in life is affected more by family members than by friends, religious faith, or personal growth. This does not mean the social capital gained by one’s networks and experiences is unimportant in shaping identity. If one does not explore opportunities in love, friendship, or a variety of vocational experiences, then they may not establish clearly delineated goals or interests, which are paramount to in-depth career exploration and thus more advanced identity statuses.

Third, the adaptive nature of career flexibility may be curvilinear in nature. In other words, there may be an optimal level of flexibility and once surpassed an individual may reconsider goals, values and interests too frequently, leading to low levels of adaptability. If so, then it may be that not all elements of VISA are linear predictors of career adaptability. Future research should further explore the associations between career reconsideration dimensions (i.e., career self doubt, career flexibility) and career adaptability.

Fourth, recent research has argued that there may be several different profiles, or classes, of helicopter parenting (Padilla-Walker et al., 2021; Hwang et al., 2022). Additionally, the numbers of profiles and prevalence of each profile, differs between mothers and fathers (Padilla-Walker et al., 2021). Furthermore, the authors argue that helicopter parenting may not be a unique parenting style, but rather a series of tactics or behaviors that occur in tandem with other parenting styles. Future research should explore if different profiles have differential effects on career adaptability and identity in emerging adults that are enrolled in college/university as well as those who are not enrolled.

Fifth, this sample was comprised of college and university educated students in Canada. It is not clear if current findings can be generalized to emerging adults who do not attend college or university. Thus, it may be that our sample captured a particularly privileged sample. Future work should explore the effects of helicopter parenting on identity and adaptability in marginalized or at risk emerging adults particularly those that have disconnected from educational or labor markets. This sentiment has been noted by others (Schwartz, 2016; Lee and Kang, 2018).

Sixth, this research does not adequately address any cross-cultural variation in emerging adult views toward helicopter parenting. While a growing body of literature supports the pervasiveness of helicopter parenting as a global phenomenon (e.g., Australia- Locke et al., 2012; Korea- Kwon et al., 2016; Scharf et al., 2017; Leung and Shek, 2018; Cui et al., 2019; Hwang et al., 2022), the current study is not comparative, and therefore further research with more diverse ethnic groups is warranted to confirm our findings in regard to the career outcomes of identity and adaptability. For example, research indicates that Chinese students report higher rates, but not greater strength, of helicopter parenting compared to American students (Hwang et al., 2022). This may in part be due to the role of traditional Confucian norms around parent-child interactions, such as simultaneous high levels of control and intense support (Lee and Kang, 2018), often well into adulthood (Kwon et al., 2016). Thus, Chinese students living in China, as well as those living in other countries but remain less acculturated to local customs, may anticipate and perhaps even expect to be exposed to helicopter parenting behaviors. Future research should explore the effects of parent-child interactions on career adaptability and identity in students from Confucian countries like China or Korea.

## Conclusion

If helicopter parenting is on the rise, as popular opinion and the nascent research suggest, it presents challenges for educators, employers and emerging adults as career seekers. This study focused on the latter group of individuals, those 18–25 years of age in the formative years of development. A significant transition during these formative years is that of the school-to-work transition. Flexible and insecure work arrangements have rendered personal agency (e.g., identity and adaptability) essential for individuals transitioning from educational to work settings. This research adds to the growing literature on helicopter parenting by exploring it’s effects on identity and adaptability during emerging adulthood. Findings indicate that this contemporary form of overparenting may detrimentally affect the career development of emerging adults by facilitating lower levels of career adaptability and in-depth exploration, as well as higher levels of career self-doubt. Furthermore, it was found that contemporary emerging adults with helicopter parents are more likely to be in the vocational identity status of searching moratorium, and this finding may add further explanation to some of the developmental delays being reported during emerging adulthood (e.g., marriage, childbirth, and stable employment). A better understanding of how helicopter parenting affects such developmental delays leads to better informed policy in both academic and work settings, as well as more impactful counseling practices for both parents and adult children.

## Data availability statement

The raw data supporting the conclusions of this article will be made available by the authors, without undue reservation.

## Ethics statement

The studies involving human participants were reviewed and approved by the University of Guelph. The patients/participants provided their written informed consent to participate in this study.

## Author contributions

JL contributed to the conception of the manuscript, the methodological design, the curation of data, the analysis of data, drafted the manuscript, critically revised and edited, and gave final approval. SL contributed to the conception of the manuscript, the methodological design, the curation of data, critically revised and edited, and gave final approval. Both authors contributed to the article and approved the submitted version.
